# Community-level risk factors for temperature-related mortality in France

**DOI:** 10.1097/EE9.0000000000000414

**Published:** 2025-09-09

**Authors:** Hicham Achebak, Pierre Masselot, Elisa Gallo, Zhao-Yue Chen, Joan Ballester, Grégoire Rey, Antonio Gasparrini

**Affiliations:** a Inserm, France Cohortes, Paris, France; b ISGlobal, Barcelona, Spain; c Environment & Health Modelling (EHM) Lab, Department of Public Health Environments and Society, London School of Hygiene & Tropical Medicine (LSHTM), London, United Kingdom

## Abstract

**Background:**

Vulnerability to nonoptimal temperatures varies from one geographical location to another, but the contextual factors accounting for these spatial differences are still poorly understood. We aimed to identify the community-level characteristics contributing to geographical disparities in heat-related and cold-related mortality risk in France.

**Methods:**

We conducted a country-wide analysis using data on all-cause mortality, temperature, and contextual characteristics across 1,967 pseudo-cantons in France between 2004 and 2019. We first estimated the daily temperature-mortality association in each pseudo-canton using a time-series quasi-Poisson regression in combination with distributed lag nonlinear models, and then we fitted univariable and multivariable multivariate meta-regression models to assess the effect modification of the contextual factors on heat-related and cold-related mortality risk.

**Findings:**

Over the 16-year study period, metropolitan France recorded 8,807,376 deaths out of an average population of 63·2 million inhabitants, which corresponds to an average annual mortality rate of 8.7 per 1,000 people. The country-level percent change (%CR) in mortality risk at the 1st and 99th daily temperature percentiles versus the minimum mortality temperature was, respectively, 31.2% (95% CI = 29.0, 33.5) and 11.0% (95% CI = 9.4, 15.5). The mortality risk associated with low temperatures was not modified by any of the contextual factors considered in the study, while the mortality risk associated with high temperatures was independently modified by NO_2_ pollution. Communities exposed to high levels of NO_2_ (i.e., cities or urban areas) had increased mortality risk from heat.

**Interpretation:**

This study suggests that urban areas in France are more vulnerable to heat, compared to rural communities, and that this disparity is probably driven by air pollution (NO_2_) and urban heat island. Reducing air pollution and mitigating urban heat island should be at the forefront of adaptation strategies to prevent heat-related health impacts.

**What this study adds**

Vulnerability to nonoptimal temperatures varies from one geographical location to another, but the contextual factors accounting for these spatial differences are still scarcely understood. The present study contributes to the literature by assessing the community-level factors contributing to geographical disparities in heat-related and cold-related mortality risk in France. It benefits from highly diversified climate and socioeconomic conditions, as well as high-resolution data, improving statistical power to detect effect modification. This information is crucial for local and national health authorities to design effective adaptation strategies to nonoptimal temperatures.

## Introduction

Air temperature is one of the major environmental determinants of human health. Every year, around five million people die prematurely because of exposure to nonoptimal temperatures, which accounts for 9.4% of all deaths globally.^1^ Nowadays, only a tenth of temperature-related mortality burden is attributed to heat,^1^ defined as days with temperatures warmer than the minimum mortality temperature (MMT), because of the frequency of hot days is smaller than that of cold days, though this situation might reverse in the future with the rise in baseline temperatures due to climate change.^2,3^

Vulnerability to nonoptimal temperatures varies from one geographical location to another,^4^ and hence identifying the community-level factors accounting for these spatial differences is an essential requirement for effective adaptation strategies minimizing the health impacts of low- and high- high-temperatures. Previous research addressing this issue found several contextual characteristics exacerbating (poor air pollution, aging, high population density, high deprivation and income inequality, and low education)^5–8^ or attenuating (more green spaces and high prevalence of air conditioning)^9–11^ heat-related mortality, although some of these associations were not consistent across all the studies. In contrast, there was limited effect modification of cold-related mortality by community-level environmental, demographic, and socioeconomic variables.^5,12,13^

However, most of these investigations focused on cities or coarse regions, providing scarce evidence for other types of settlements, such as peri-urban and rural areas.^8,14^ Second, the number of locations and contextual factors analyzed was relatively low. Third, as many indicators exhibited collinearity, former studies only performed univariable models (i.e., one indicator as an explanatory variable), and therefore, independent effects of the predictors could not be isolated. Increasing the sample size can increase the power in the presence of multicollinearity by adding more variation to the data, making it easier to distinguish between the contributions of different predictors.

The present study aimed to identify the community-level factors contributing to geographical disparities in heat-related and cold-related mortality risk in France. Former investigations of this kind in the country by Pascal and colleagues^7,9^ were conducted only in the Paris region and analyzed a limited number of contextual factors as effect modifiers of the heat-mortality association. Our study benefits from highly diversified climate and socioeconomic conditions, as well as high-resolution data, improving statistical power to detect effect modification. This information is crucial for local and national health authorities to design effective adaptation strategies to nonoptimal temperatures.

## Methods

### Data sources

The present study was conducted in metropolitan France (i.e., mainland France plus the island of Corsica) between 1 Jan 2004 and 31 Dec 2019, at the canton-ou-ville (or pseudo-canton) level. A pseudo-canton represents a grouping of one or more contiguous communes (or municipalities ~36,000 on average). In contrast, the three largest municipalities in France (i.e., Paris, Marseille, and Lyon) were analyzed at the arrondissements municipaux (or municipal districts) for consistency in area-level population. In total, the study included 1,967 pseudo-cantons, among which 45 corresponded to municipal districts.

Individual mortality records with identifier of municipality (or district) of residence and date of death were provided by the Epidemiology Center on Medical Causes of Death (CépiDC) of the National Institute of Health and Medical Research (Inserm) and then aggregated as pseudo-canton-specific daily series of all-cause mortality counts.

Daily mean observations of 2-meter air temperature (°C) and relative humidity (%) on 10 × 10 km grid cells across metropolitan France were extracted from the E-OBS database^15,16^ (version 30·0e), while daily mean concentrations of various air pollutants (particulate matter ≤ 10 [PM_10_], particulate matter ≤ 2·5 [PM_2·5_], nitrogen dioxide [NO_2_], and maximum 8-h average ground-level ozone [O_3_]) were estimated using a quantile machine learning model at a spatial resolution of 10 × 10 km, as described elsewhere.^17^ We obtained the corresponding pseudo-canton-specific daily weather and air pollution series by computing the area-weighted average of the values of the grid cells intersecting the pseudo-canton boundaries, with weights proportional to the intersection areas.

Lastly, data on environmental, demographic, and socioeconomic factors were collected for all the municipalities in France and then aggregated at the pseudo-canton level to calculate contextual indicators potentially linked with spatial disparities in vulnerability to nonoptimal temperatures (Table 1). These ecological indicators included measures of climate, air pollution, degree of urbanicity or rurality, housing characteristics, population aging, social isolation, education, socioeconomic conditions, and voting behavior. Details on data sources and a description of contextual indicators are provided in the Table S1; https://links.lww.com/EE/A368.

**Table 1.** Descriptive statistics for contextual indicators

Indicator	Min	Mean	Median	Max	IQR
Climate					
Temperature (°C)	1.7	12.0	11.8	16.3	1.6
Relative humidity (%)	64.4	77.4	78.4	84.8	4.7
Air pollution					
Particulate matter ≤10 µm (µg/m^3^)	12.8	19.8	19.3	37.5	3.9
Particulate matter ≤2.5 µm (µg/m^3^)	8.9	13.4	13.0	37.1	2.3
Nitrogen dioxide (µg/m^3^)	8.6	12.7	11.6	21.7	2.8
Ground-level ozone (µg/m^3^)	67.9	75.2	74.3	91.5	4.9
Land cover					
Artificial surface (%)	0.2	20.8	7.7	100.0	25.0
Type of settlement					
Population density (people/km^2^)	8	1,208	133	40,742	577
Rural population (%)	0.0	52.9	55.4	100.0	92.1
Housing conditions					
Houses built before 1971 (%)	4.7	43.6	44.1	91.2	18.0
Houses with central heating (%)	7.3	55.4	54.6	94.0	24.1
Home ownership (%)	21.7	64.7	68.7	86.9	16.7
Population aging					
People over 64 years (%)	6.6	20.4	19.9	44.3	6.7
Social isolation					
People over 64 years living alone (%)	20.1	32.0	31.3	52.0	6.2
Education					
People over 24 years without primary education (%)	5.4	15.7	15.3	38.9	6.2
Socioeconomic conditions					
Median income by consumption unit (€)	7,355	19,411	18,725	45,776	3,652
Households with 2 or more cars (%)	1.8	40.6	43.2	65.9	14.2
Unemployment rate (%)	3.3	10.7	9.9	29.6	4.0
Voting behavior					
Votes for the right (%)	25.2	52.3	52.4	80.8	14.7

Summary statistics are based on the average value of the contextual indicators across all the pseudo-cantons.

IQR indicates interquartile range.

### Statistical analysis

A two-stage analysis was adopted in this multi-location study:

In the first stage, we estimated the daily temperature-mortality association across pseudo-cantons using a time-series quasi-Poisson regression in combination with distributed lag nonlinear models (DLNM).^18^ The regression model included: (1) an intercept, (2) a categorical variable of day of the week to account for intra-weekly variation in mortality, (3) a natural cubic B-spline of time with 8 degrees of freedom (DF) per year to control for seasonal and long-term trends in mortality, (4) a natural cubic B-spline with 2 DF to adjust for same-day relative humidity, and (5) a cross-basis function produced by DLNM to characterize the nonlinear and delayed effects of temperature on mortality. Consistent with well-tested parametrisation used in many previous studies,^1,4^ the natural cubic B-spline describing the exposure-response function in the cross-basis was modeled with three internal knots placed at the 10th, 75th and 90th percentiles of the daily temperature distribution, while the natural cubic B-spline representing the lag-response function was modeled with an intercept and three internal knots placed at equally spaced values in the log scale and a lag period extending up to 3 weeks. The sets of 20 coefficients (i.e., 4 [exposure-response function] × 5 [lag-response function]) obtained from the cross-basis were then reduced to sets of four coefficients of unidimensional B-splines that model the overall cumulative exposure-response association (i.e., cumulation of the effect of temperature over the lag dimension).

In the second stage, we fitted univariable and multivariable multivariate meta-regression models to assess the effect modification of contextual characteristics on the temperature-mortality relationship.^19,20^ The meta-regression model can algebraically be written as:

Y_i_ = α + β_j_X_ij_ + δ_i_ + ϵ_i_,

δ_i_ ∼ N(0, τ_i_), ϵ_i_ ∼ N(0, S_i_),

where Y_i_ denotes the vector of spline parameters representing the temperature-mortality association in pseudo-canton _i_ (N = 1,967); X_ij_ is the contextual characteristic for which effect modification is estimated, along with other potentially confounding characteristics with fixed effect coefficient vector β_j_; δ_i_ is a random intercept having unstructured (co)variance matrices τ; and ϵ the error term distributed with pseudo-canton (co)variance matrices S_i_. To summarize the effect of each meta-predictor on vulnerability to heat and cold, we used the fitted meta-regression model to predict the relative risk (RR) of mortality associated with temperatures (i.e., temperature-mortality curves [see Figure S1; https://links.lww.com/EE/A368]) for the 90th and 10th percentiles of the meta-predictors, keeping others at their average value. Using the MMT as a reference, we then took, respectively, the RR of mortality at the 1st and 99th temperature percentiles from the two predicted temperature-mortality curves and calculated the ratio between both RR (RRR = exp(log[RR_90th percentile meta-predictor_] – log[RR_10th percentile meta-predictor_])), which was then transformed into a percentage change in risk (%CR) of mortality (%CR = [RRR – 1] × 100). A positive value of %CR indicated an increased risk of mortality from cold or heat associated with the contextual variable, and vice versa for a negative value of %CR. Note that when the empirical confidence interval computed (see details in Text S1; https://links.lww.com/EE/A368) for %CR did not contain the null hypothesis value (zero), then the result was considered statistically significant. Lastly, the final multivariate meta-regression model was used to derive the best linear unbiased prediction of the overall cumulative exposure-response associations in each pseudo-canton.

All statistical analyses were performed with R software (version 4·4·2), using the dlnm and mixmeta packages.

## Results

### Descriptive analysis

Over the 16-year study period (2004–2019), metropolitan France recorded 8,807,376 deaths out of an average population of 63.2 million inhabitants, which corresponds to an average annual mortality rate of 8.7 per 1,000 people. The 0.02% of all recorded deaths (2,075 in absolute terms) had no identifier of municipality of residence, and therefore, were excluded from the analysis. The average daily number of deaths was 0.8 (inter-pseudo-canton range 0.1–10·2), while the average daily exposure to temperature and relative humidity was, respectively, 12.0 °C (range 1.7–16.3) and 77·4% (range 64.4–84.8). The temperature and humidity series had no missing values.

Summary statistics for the contextual indicators are reported in Table 1. The inter-location range of the variables (i.e., the difference between the maximum and minimum values) highlighted remarkable differences in environmental, demographic, and socioeconomic conditions across the country. For example, the average exposure to PM_2·5_ ranged from 8.9 µg/m^3^ to 37.1 µg/m^3^; the proportion of population over 64 years ranged from 6.6% to 44.3%; the percentage of population without completing primary school ranged from 5.4% to 38.9%; and the median income by consumption unit from 7,355€ to 45,776€. Moreover, some variables showed a clear geographical pattern, with values increasing (e.g., relative humidity) or decreasing (e.g., temperature, O_3_) from South to North (Figure S2; https://links.lww.com/EE/A368).

### Univariable regression analysis

**Figure 1.**
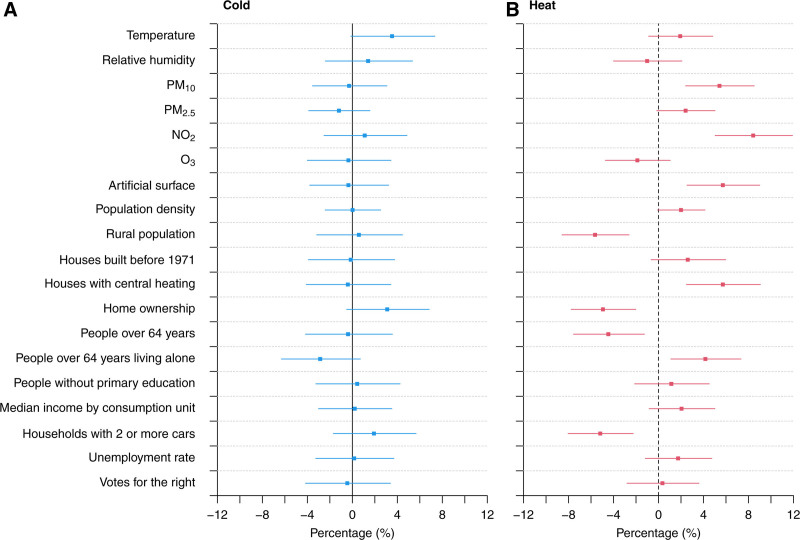
Effect modifiers of heat- and cold-related mortality. The figure represents the %CR of mortality at the 1st (cold) and 99th (heat) percentiles of daily temperature between the 10th and 90th percentiles of each contextual indicator from univariable meta-regression models. Error bars represent the 95% confidence interval. Numerical information is reported in Table S2; https://links.lww.com/EE/A368.

Figure 1 shows the results from univariable meta-regression models, namely the %CR of mortality at the 1st (i.e., cold; Figure 1A) and 99th (i.e., heat; Figure 1B) percentiles of daily temperature (cumulated within lag 0–21) associated with contextual variables. There was little evidence of an association for cold-related mortality risk with any of the contextual factors. Conversely, there was evidence that the heat-related mortality risk was: (1) positively associated with some air pollutants (i.e., PM_10_ and NO_2_) and the percentage of artificial surfaces, houses with central heating, and people over 64 years living alone, and (2) negatively associated with the proportion of rural population, home ownership, population over 64 years, and households owning two or more cars. However, other predictors were clearly close to statistical significance, such as PM_2·5_, population density, proportion of houses built before 1971, income, and unemployment rate. Moreover, it is important to point out that the community-level variables exacerbating the mortality risk of heat were positively correlated with urbanicity (e.g., using NO_2_ as a proxy of urbanicity) while the converse was true for the variables lowering the mortality risk of heat (Figure S3; https://links.lww.com/EE/A368). The estimates were found to be robust in sensitivity analyses (Figure S4; https://links.lww.com/EE/A368).

### Multivariable regression analysis

**Figure 2.**
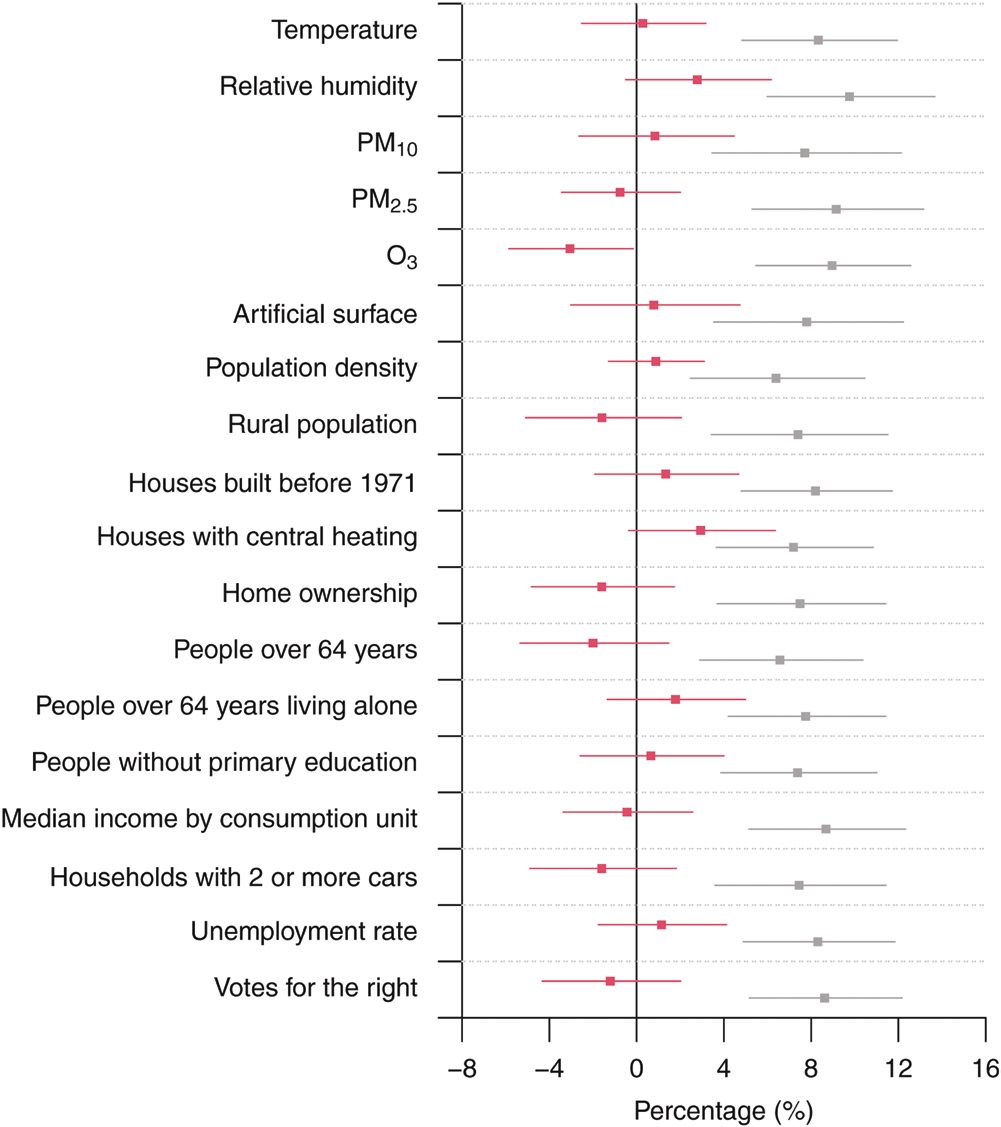
Effect modifiers of heat-related mortality risk after adjustment for nitrogen dioxide. The figure represents the %CR of mortality at the 99th percentile of daily temperature between the 10th and 90th percentiles of each contextual indicator from bivariable meta-regression models. Red squares represent the %CR of mortality associated with each contextual indicator after adjustment for nitrogen dioxide, while gray squares represent the %CR of mortality associated with the share of nitrogen dioxide after adjustment for each contextual indicator. Error bars represent the 95% confidence interval. Numerical information is reported in Table S3; https://links.lww.com/EE/A368.

Figure 2 shows the results from bivariable meta-regression models, namely the %CR of mortality at the 99th (i.e., heat) percentile of daily temperature associated with each one of the contextual variables after introducing the level of NO_2_ as a second meta-predictor in univariable meta-regression models (Figure 1B). When accounting for NO_2,_ none of the contextual factors continued to have a statistically significant effect modification on the heat-mortality relationship (red square with error bar), while the contribution of NO_2_ remained statistically significant in all bivariable models (gray squares with error bar). Note that we introduced the level of NO_2_ as a second meta-predictor in bivariable models because this variable exhibited the strongest modifying effect on heat-mortality association in univariable models, and because it is a good proxy of the degree of urbanicity/rurality in our database.

### Mapping of the risk

**Figure 3.**
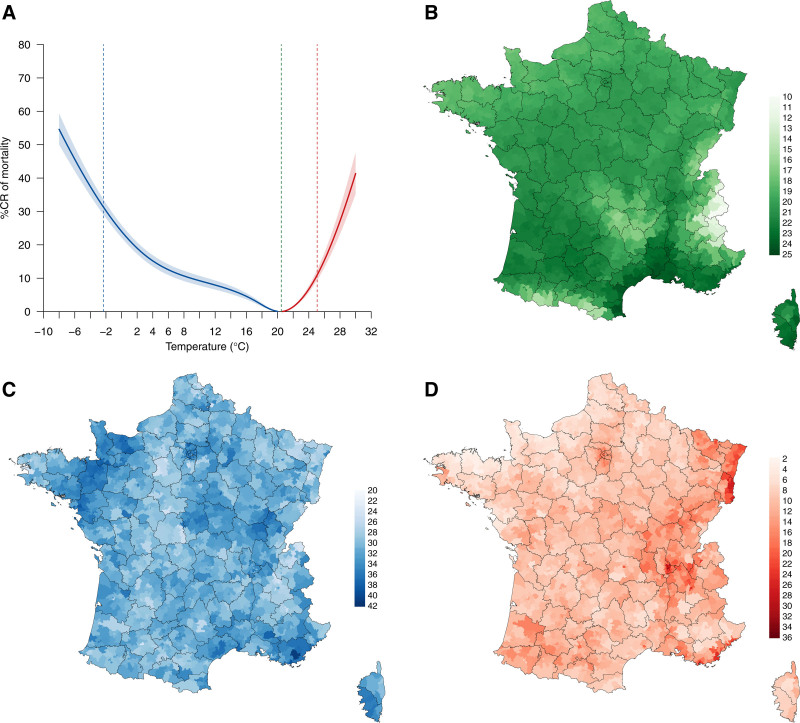
Relationship between daily mean temperature and mortality in France. In panel (A) the %CR of mortality is cumulated within lag 0–21 and centered at the MMT. Panels (C) and (D) represent the %CR of mortality risk at the 1st and 99th percentiles of daily temperature versus the MMT, respectively. Black outlines in the maps represent the 96 Departments of metropolitan France.

Figure 3A displays the overall temperature-mortality relationship for France as a whole, resulting from the pooling of pseudo-cantons estimates through a meta-regression model that included NO_2_ as the sole meta-predictor. The association drew a classical inverse J-shape curve, with MMT at 20.5°C (corresponding to the 90th temperature percentile) and increased risks at both low- and high-temperatures. The %CR of mortality at the 1st (cold) and 99th (heat) temperature percentiles was, respectively, 31.2% (95% CI = 29.0, 33.5) and 11·0% (95% CI = 9.4, 15.5). Additionally, Figures 3B,C map, respectively, the MMT and the %CR of mortality at the location-specific 1st and 99th temperature percentiles from best linear unbiased predictions. There was a strong positive correlation between the MMT and average temperature (r = 0.92; Figure S5; https://links.lww.com/EE/A368), with the lowest MMT values occurring in high-altitude areas such as the Pyrenees, the Massif Central, or the Alps (Figure 3B). Moreover, Northern France (except the extreme east) had the lowest mortality risks from heat, while the north-east and central-east regions (i.e., Alsace, Lorraine, Franche-Comté, and Rhône-Alpes) emerged as the main hotspots of heat vulnerability in the country (Figure 3D). Finally, no clear geographical pattern was observed for cold-related mortality risks (Figure 3C).

## Discussion

The main purpose of this country-wide study was to assess the role that community-level characteristics play in the geographical patterns of heat- and cold-related mortality risks across France, and in this way provide valuable information to guide climate change health adaptation in the country and beyond. The mortality risk associated with low temperatures was not modified by any of the contextual factors considered in this analysis, while the mortality risk associated with high temperatures was independently modified by NO_2_ pollution. Communities exposed to high levels of NO_2_ (i.e., cities or urban areas) had increased mortality risk from heat.

The interpretation of the effect modification of heat-related mortality by NO_2_ could be two-fold. First, NO_2_ pollution might be amplifying the mortality associated with heat through synergistic effects. That has not only been confirmed in previous studies,^21,22^ but also in our first-stage time-series regression analysis, interacting the effect of temperature with NO_2_ concentrations, which revealed a stronger heat-mortality association on days with elevated levels of NO_2_ (Figure S6; https://links.lww.com/EE/A368). These strongly suggest higher vulnerability to heat for populations that are also exposed to higher levels of NO_2_. Second, NO_2_ is an air pollutant that is closely associated with traffic-related pollution in urban areas, and therefore, it can serve as a reliable proxy for the degree of urbanization and urban heat island (UHI).^23,24^ Cities experience higher temperatures than the surrounding rural areas because of the UHI phenomenon, and this overexposure to heat, which is not well captured by our coarse temperature data, might also be exacerbating the mortality risk from high temperatures. UHI is a modifiable risk factor that is being intensified by increasing urbanisation and rising temperatures associated with climate change.^25^ Hence, UHI mitigation strategies such as increasing the vegetative cover (which cools down temperatures through shading and evapotranspiration),^26^ incorporating solar-reflective materials (on walls, roofs, and pavements)^27^ and improving building energy efficiency (e.g., emitting less heat [most of which from air conditioning use] to the environment),^28^ are a pressing need to minimize the health risks posed by UHI.

Previous research addressing vulnerability to high temperatures across urban and rural areas yielded mixed results. Some studies reported larger heat-related mortality risk in urban areas (e.g., England and Wales,^14^ Spain,^29^ and Switzerland^30^), others showed greater risk in rural areas (e.g., China^31,32^ and South Korea^33^), and some others found a similar risk for rural and urban communities (e.g., Mexico^6^). Generally, it appears from the available literature that, compared with urban areas, rural communities are less vulnerable to heat in more-developed countries and equally or more susceptible to heat in less-developed countries. We hypothesized that while in the former settings air pollution and UHI might be the main drivers of heat-related health impacts, in the latter settings those impacts might be depending more on nonenvironmental contextual factors. In low- and middle-income countries, rural areas might be lagging far behind urban areas in terms of socioeconomic development (e.g., access to health services).

In this study, demographic and socioeconomic factors had no clear role on heat- and cold-related mortality risks, although this unexpected finding might not be extrapolated to other geographical scales, such as in intra-urban settings. Our results for cold are in line with those reported in multi-city studies across different countries, which found little evidence of effect modification of cold-related mortality by demographic and socioeconomic characteristics.^5,12^ This situation could partly respond to more complex mechanisms through which low temperatures affect health, such as infectious diseases. However, a recent study conducted in Spain found lower vulnerability to cold-related mortality in regions with higher prevalence of heating (colder regions),^34^ while in Switzerland, higher deprivation and longer travel time to healthcare increased susceptibility to cold, both in urban and rural areas.^30^ Moreover, in some investigations, several demographic and socioeconomic factors were found to exacerbate (e.g., aging, high deprivation, high income inequality, and low education)^5–7^ or attenuate (e.g., high prevalence of air conditioning)^11^ the risk of mortality from heat, although the contribution of these variables was not always consistent across all the geographical scales of analysis and types of settlement (i.e., urban, peri-urban/rural, rural).^30^

This study had strengths and limitations. On the one hand, we used country-wide high-quality high-resolution health and meteorological data, which allowed us to accurately characterize the geographical pattern of heat- and cold-related mortality risk throughout France, making use of the most advanced methods in environmental epidemiology. We also expanded the previous literature by assessing a wider range of community-level factors potentially explaining the spatial variation in vulnerability to high and low air temperatures. On the other hand, regional data on air conditioning (AC) prevalence were not available, which could influence the risk of mortality from heat. However, the prevalence of AC, which is, on average, still low in France (~ 25% of French households had AC in 2020^35^), is probably correlated with some of the many contextual variables included in the analysis (e.g., temperature, income). Moreover, we did not examine the potential nonlinear effects of community-level factors on heat- and cold-related risks, as this can be very complex and imprecise. And finally, our findings might not be applicable to developing countries.

In conclusion, this study suggests that urban areas in France are more vulnerable to heat, compared with rural communities, and that this disparity is probably driven by air pollution (NO_2_) and UHI. Reducing air pollution and mitigating UHI should be at the forefront of adaptation strategies to prevent heat-related health impacts.

## Conflicts of interest statement

The authors declare that they have no conflicts of interest with regard to the content of this report.

## Supplementary Material


