# Supplementary material for: Community-level risk factors for temperature-related mortality in France
Source: Environ Epidemiol. 2025 Sep 9;9(5):e414. doi: 10.1097/EE9.0000000000000414 (PMC12422778; doi:10.1097/EE9.0000000000000414)
Supplement: Supplementary file 1 [file ee9-9-e414-s001.pdf]

# *Supplementary Material*

# Community-level risk factors for temperature-related mortality in France

*Hicham Achebak[1-2], Pierre Masselot[3], Elisa Gallo[2], Zhao-Yue Chen[2], Joan Ballester[2], Grégoire Rey[1†], Antonio Gasparrini[3†]*

[1]Inserm, France Cohortes, Paris, France; [2]ISGlobal, Barcelona, Spain; [3]Environment & Health Modelling (EHM) Lab, Department of Public Health Environments and Society, London School of Hygiene & Tropical Medicine (LSHTM), London, United Kingdom.

[†]Contributed equally

Correspondence to:
Dr. Hicham Achebak
Barcelona Institute for Global Health
08003 Barcelona, Spain
**hicham.achebak@isglobal.org**

## Table of contents

Table S1. Description of contextual indicators ..... 3
Figure S1. Predictions of the overall cumulative exposure-response association ..... 4
Text S1. Computation of confidence interval for percentage change in risk (%CR) of mortality ..... 6
Figure S2. Geographical distribution of contextual indicators ..... 7
Figure S3. Correlation matrix for contextual indicators ..... 10
Figure S4. Sensitivity analyses ..... 11
Table S2. Effect modifiers of heat- and cold-related mortality ..... 13
Table S3. Effect modifiers of heat-related mortality risk after adjustment for nitrogen dioxide ..... 14
Figure S5. Relationship between temperature and minimum mortality temperature ..... 15
Figure S6. Ratio of temperature-related mortality risk between high and low air pollution days ..... 16

**Table S1. Description of contextual indicators**

| Indicator | Description | Geography | Time | Source |
|---|---|---|---|---|
| Temperature | Gridded daily mean temperature observations (°C) | 0.1deg (≈10km×10km) | 2004-2019 | E-OBS (Version 30.0e) |
| Humidity | Gridded daily mean relative humidity observations (%) | | | |
| Particulate matter ≤ 10 and ≤ 2.5 µm; nitrogen dioxide; ground-level ozone. | Gridded daily mean estimations (µg/m$^3$) | 0.1deg (≈10km×10km) | 2004-2019 | Chen, ZY. et al. *Nat Commun* 15, 2094 (2024) |
| Artificial surface | Proportion (%) of (i) Urban fabric, (ii) Industrial, commercial and transport units, and (iii) Mine, dump and construction sites | Commune (N≈36,000), Arrondissement municipal (N=45) | 2006, 2012, 2018 | Ministères Territoires Écologie Logement. CORINE Land Cover |
| Population density | People per km$^2$ | | 2009, 2014 2020 | Observatoire des Territoires Insee. Comparateur de territoires |
| Rural Population | Proportion (%) of people living in 1km×1km rural grid cells | | 2015-2019 | Insee. La grille communale de densité |
| Houses built before 1971 | Proportion (%) of houses built before 1971 | | 2006-2019 | Insee. Recensement de la population |
| Houses with central heating | Proportion (%) of houses with central heating | | | |
| Owned houses | Proportion (%) of houses in property | | | |
| Households with 2 or more cars | Proportion (%) of households with 2 or more | | | |
| People over 64 years | Proportion (%) of people aged 65 and more | | 2009, 2014, 2020 | Insee. Recensement de la population |
| People over 64 years living alone | Proportion (%) of people aged 65 and more living alone | | 2007-2020 | Insee. Recensement de la population |
| People over 24 years without primary education | Proportion (%) of people aged 24 and more without primary school diploma | | 2009, 2020 | Insee. Recensement de la population |
| Income | Median income by consumption unit (€) | | 2004-2019 | Insee. Dispositif Fichier localisé social et fiscal (Filosofi) |
| Unemployment | Unemployment rate (%) among people aged 15-64 | | 2009, 2014, 2020 | Observatoire des Territoires Insee. Comparateur de territoires |
| Voting | Proportion (%) of votes for the right | | 2007, 2012, 2017 | J Cagé et T Piketty : Une histoire du conflit politique, Le Seuil, 2023 |

**Figure S1. Predictions of the overall cumulative exposure-response association**

The figure represents the relative risk (RR) of mortality associated with daily temperature predicted from univariable meta-regression model for the 10th (red curve) and 90th (blue curve) percentiles of each contextual indicator vs the MMT. Shaded areas represent the 95% CI.

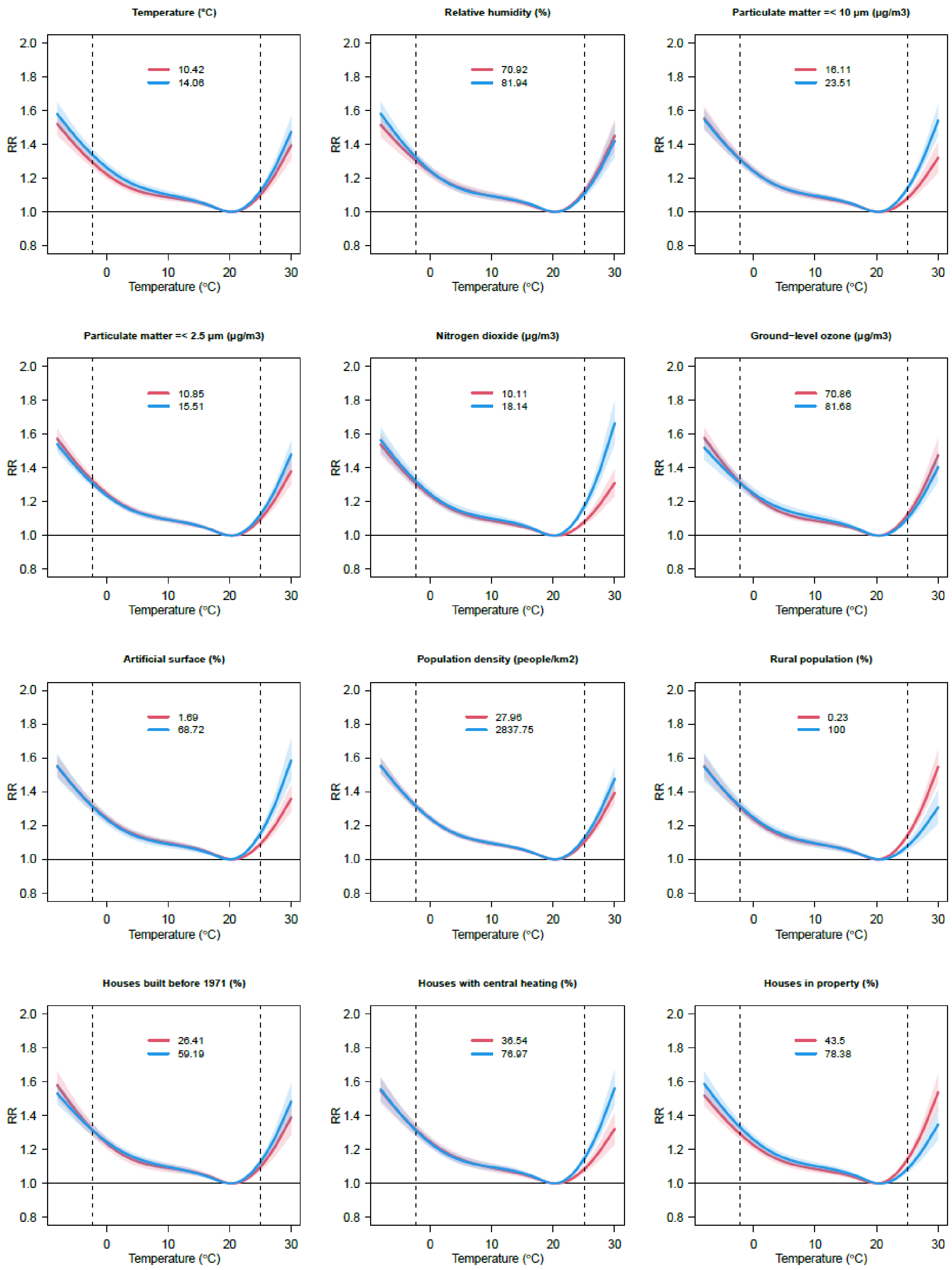

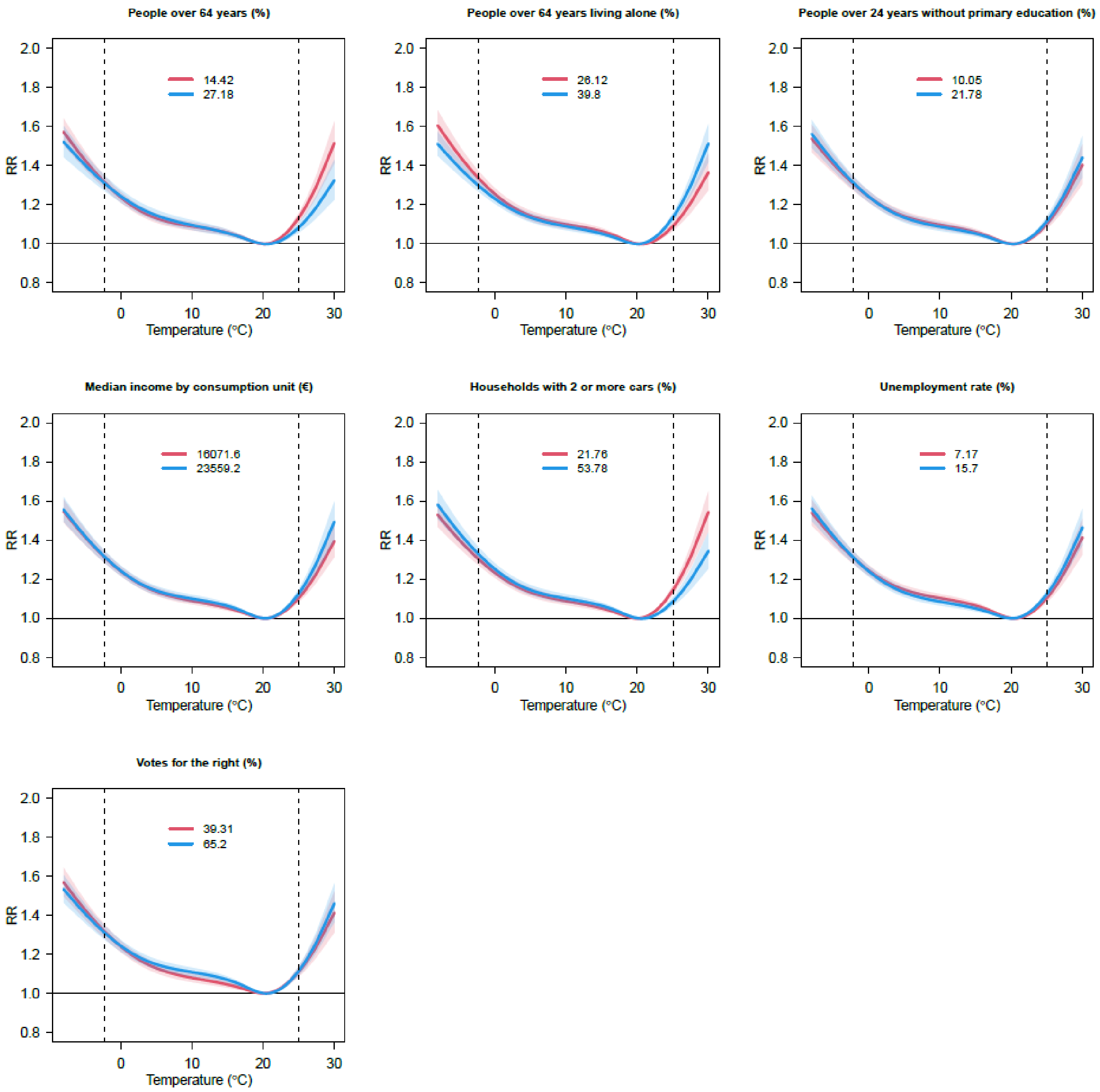
People over 64 years (%)
14.42
27.18
People over 64 years living alone (%)
26.12
39.8
People over 24 years without primary education (%)
10.05
21.78
Median income by consumption unit (€)
16071.6
23559.2
Households with 2 or more cars (%)
21.76
53.78
Unemployment rate (%)
7.17
15.7
Votes for the right (%)
39.31
65.2
RR
2.0
1.8
1.6
1.4
1.2
1.0
0.8
0
10
20
30
Temperature (°C)

**Text S1. Computation of confidence interval for percentage change in risk (%CR) of mortality**

First, we computed the log(RRR) and associated standard error (SE):

- $\log(RRR) = \log(RR_{\text{max. value meta-predictor}}) - \log(RR_{\text{min. value meta-predictor}})$
- $SE = \text{sqrt}( (SE_{\text{max. value meta-predictor}})^2 + (SE_{\text{min. value meta-predictor}})^2 )$

Then, we calculated the confidence interval for %CR as follows:

- Lower bound of CI: (exp( log(RRR) - 1.96 * SE ) – 1) x 100
- Upper bounds of CI: (exp( log(RRR) + 1.96 * SE ) – 1) x 100

**Figure S2. Geographical distribution of contextual indicators**

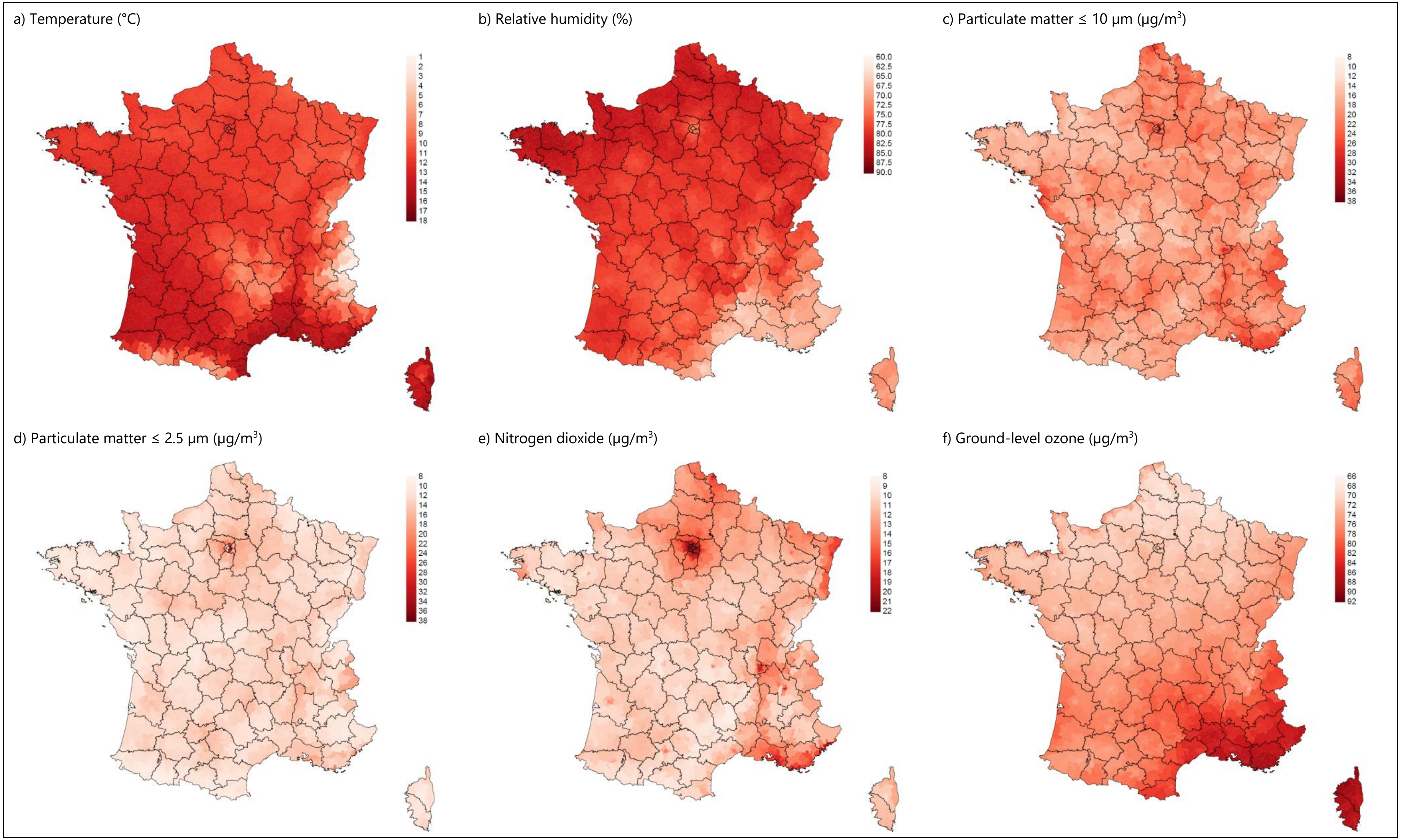

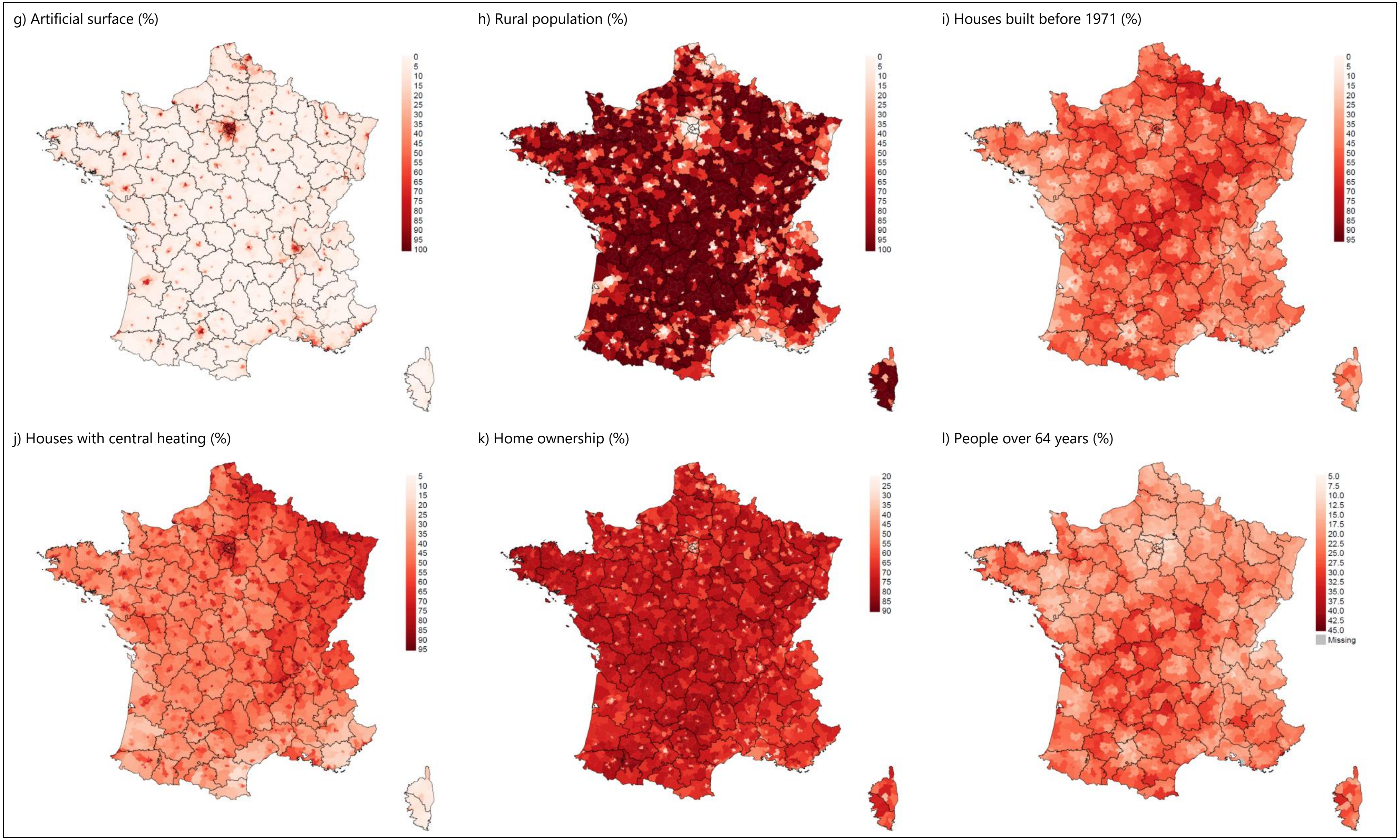
g) Artificial surface (%)
0 5 10 15 20 25 30 35 40 45 50 55 60 65 70 75 80 85 90 95 100
h) Rural population (%)
0 5 10 15 20 25 30 35 40 45 50 55 60 65 70 75 80 85 90 95 100
i) Houses built before 1971 (%)
0 5 10 15 20 25 30 35 40 45 50 55 60 65 70 75 80 85 90 95
j) Houses with central heating (%)
5 10 15 20 25 30 35 40 45 50 55 60 65 70 75 80 85 90 95
k) Home ownership (%)
20 25 30 35 40 45 50 55 60 65 70 75 80 85 90
l) People over 64 years (%)
5.0 7.5 10.0 12.5 15.0 17.5 20.0 22.5 25.0 27.5 30.0 32.5 35.0 37.5 40.0 42.5 45.0
Missing

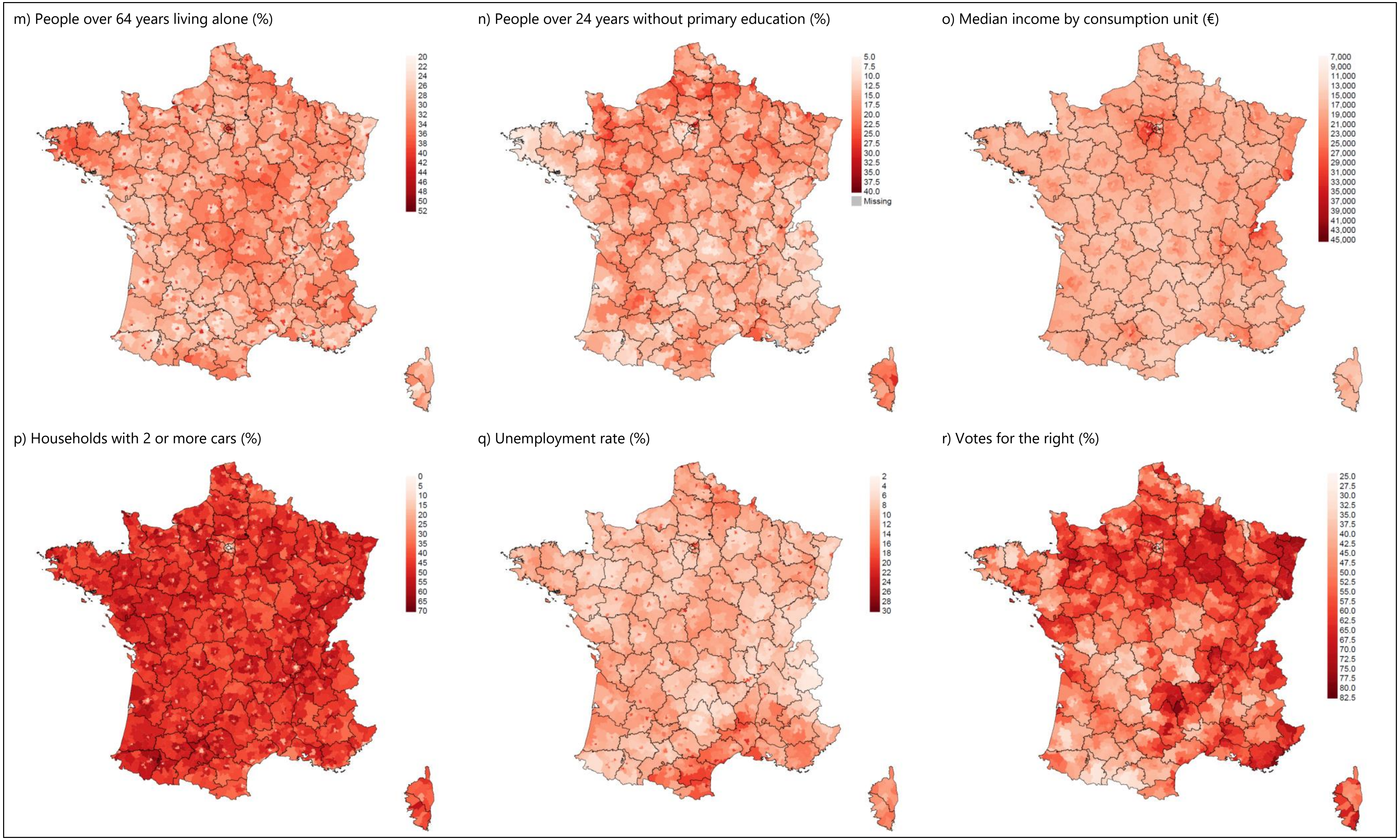
m) People over 64 years living alone (%)
20 22 24 26 28 30 32 34 36 38 40 42 44 46 48 50 52
n) People over 24 years without primary education (%)
5.0 7.5 10.0 12.5 15.0 17.5 20.0 22.5 25.0 27.5 30.0 32.5 35.0 37.5 40.0
Missing
o) Median income by consumption unit (€)
7,000 9,000 11,000 13,000 15,000 17,000 19,000 21,000 23,000 25,000 27,000 29,000 31,000 33,000 35,000 37,000 39,000 41,000 43,000 45,000
p) Households with 2 or more cars (%)
0 5 10 15 20 25 30 35 40 45 50 55 60 65 70
q) Unemployment rate (%)
2 4 6 8 10 12 14 16 18 20 22 24 26 28 30
r) Votes for the right (%)
25.0 27.5 30.0 32.5 35.0 37.5 40.0 42.5 45.0 47.5 50.0 52.5 55.0 57.5 60.0 62.5 65.0 67.5 70.0 72.5 75.0 77.5 80.0 82.5

**Figure S3. Correlation matrix for contextual indicators**

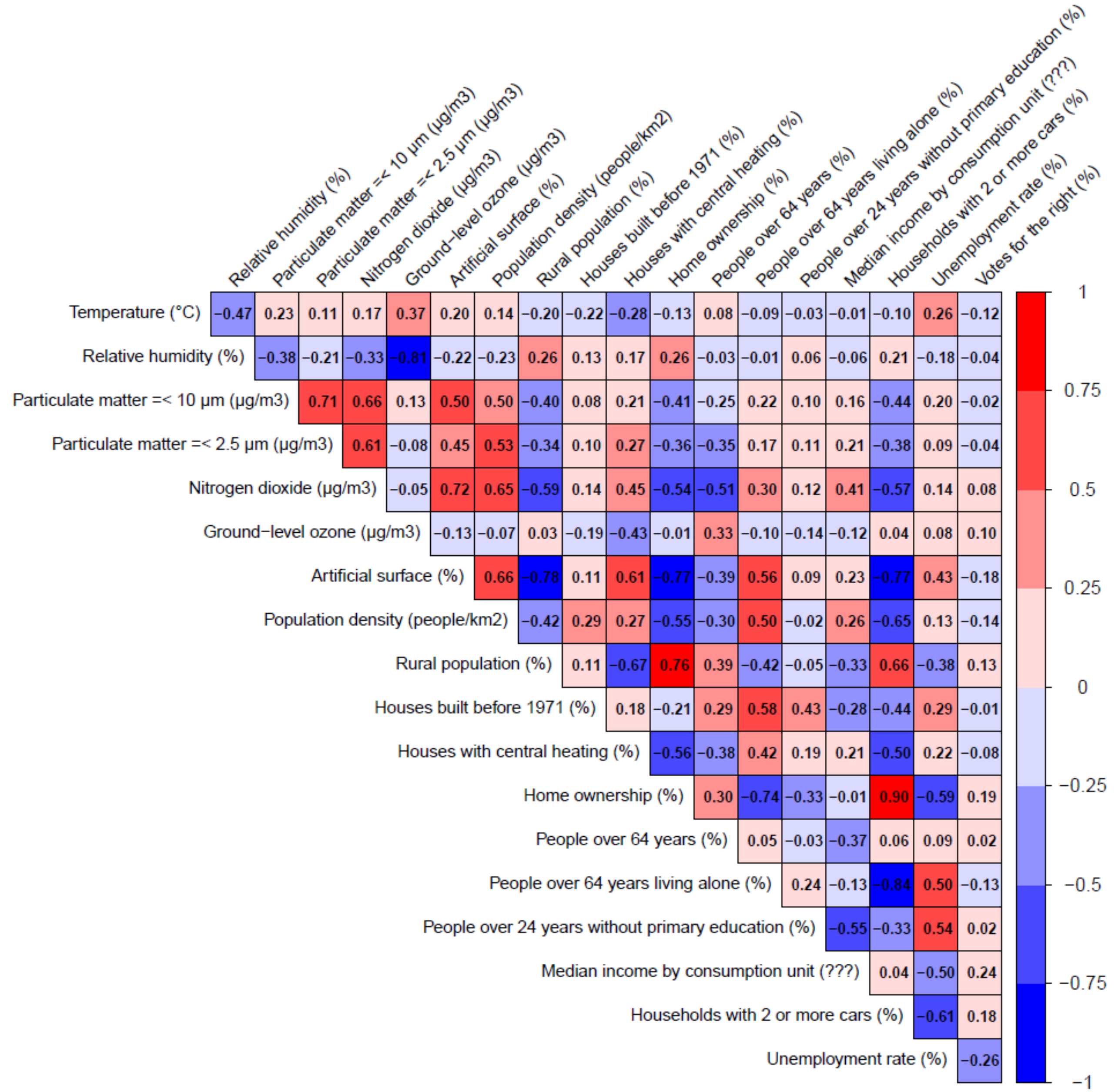

**Figure S4. Sensitivity analyses**

a) Degrees of freedom for seasonality in the 1st stage time-series regression

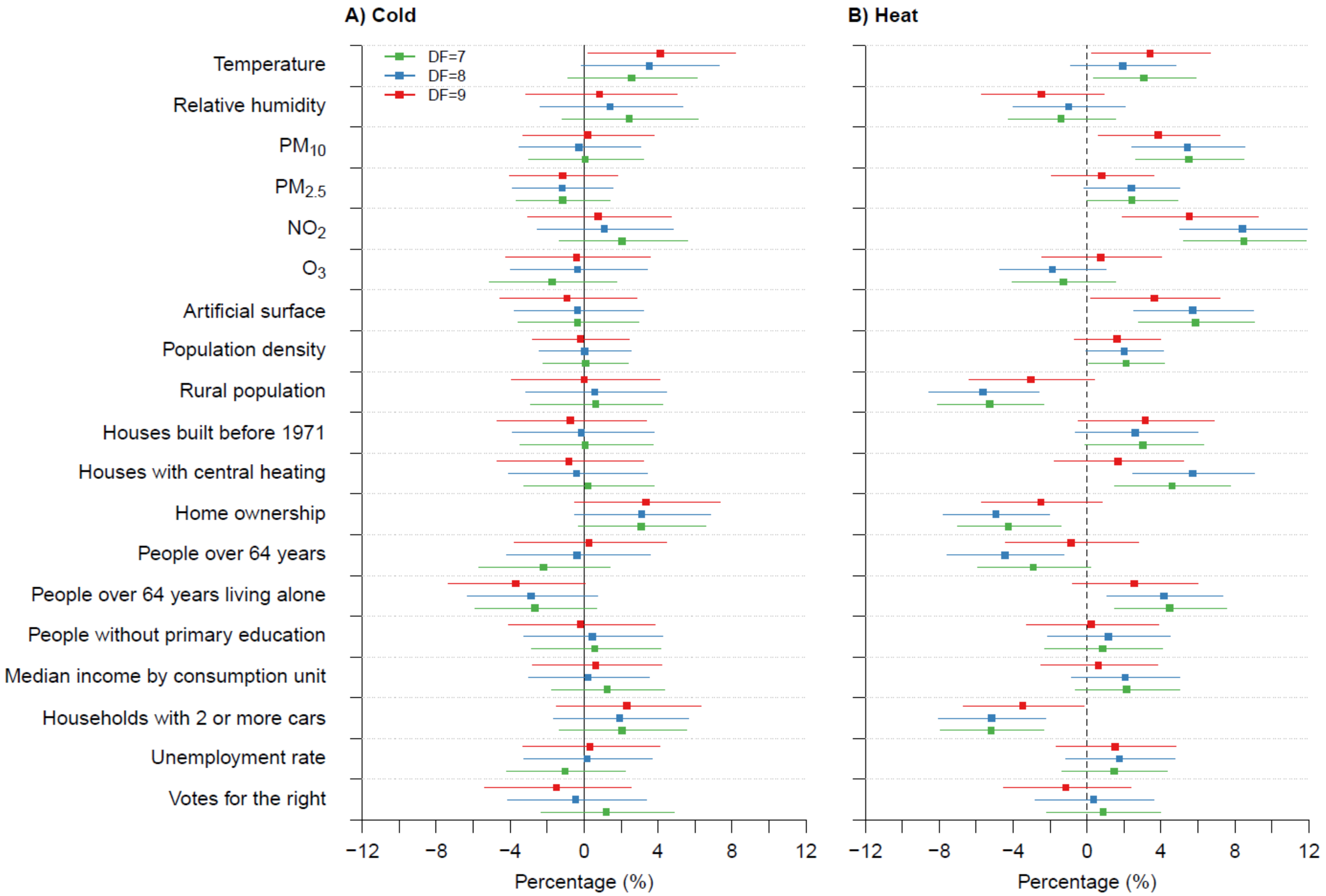

**Figure S4. Sensitivity analyses**

b) Lag days for the cross-basis of temperature in the 1st stage time-series regression

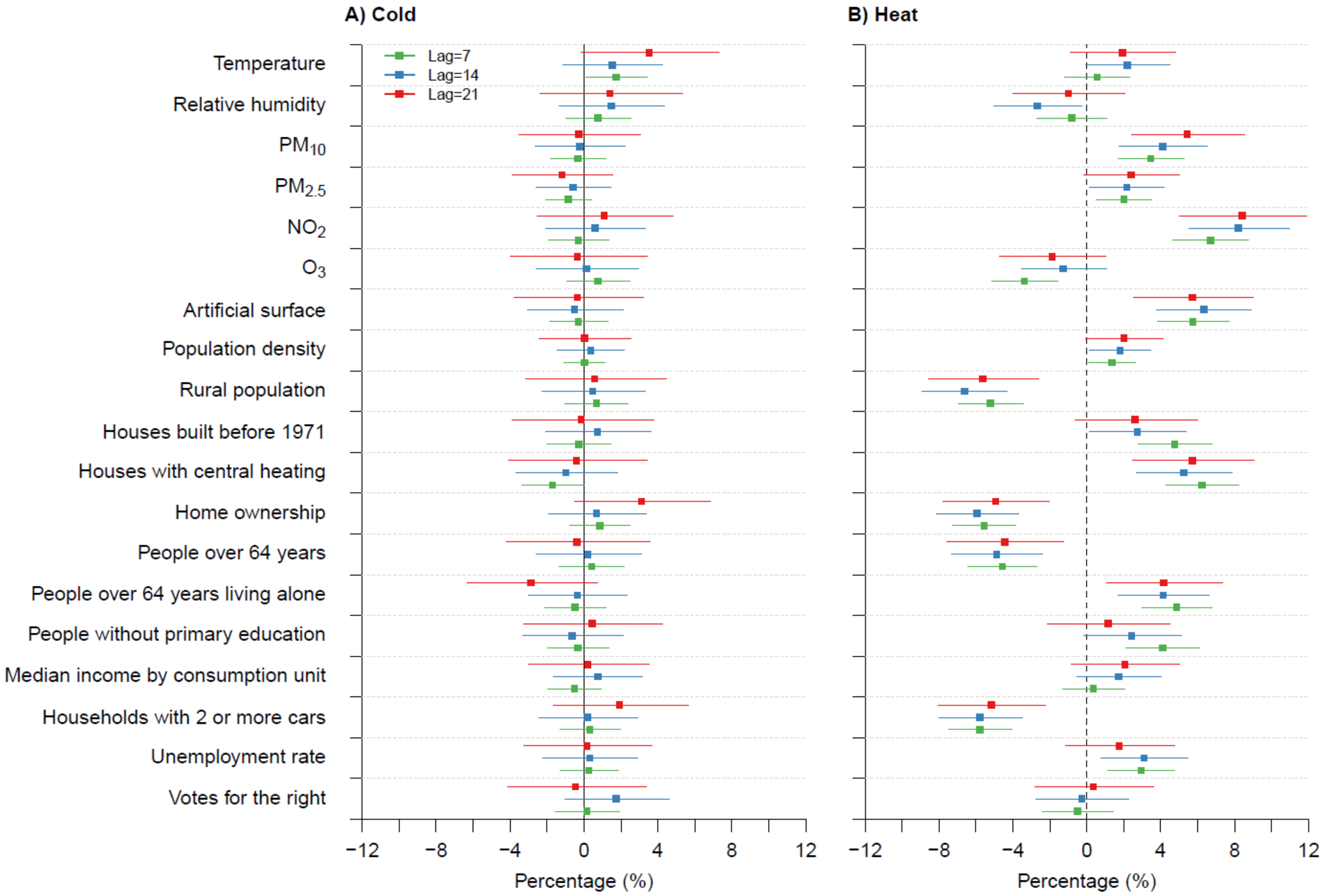

**Table S2. Effect modifiers of heat- and cold-related mortality**

The table reports the %CR of mortality at the 1st (cold) and 99th (heat) percentiles of daily temperature the 10th and 90th percentiles of each contextual indicator from univariable meta-regression models.

| | Cold | Heat |
|---|---|---|
| Temperature | 3.53 (-0.13 to 7.33) | 1.93 (-0.87 to 4.82) |
| Relative humidity | 1.4 (-2.4 to 5.35) | -1.01 (-4 to 2.08) |
| Particulate matter ≤ 10 μm | -0.29 (-3.54 to 3.07) | 5.42 (2.41 to 8.51) |
| Particulate matter ≤ 2.5 μm | -1.19 (-3.88 to 1.57) | 2.41 (-0.15 to 5.02) |
| Nitrogen dioxide | 1.1 (-2.52 to 4.85) | 8.42 (5.04 to 11.91) |
| Ground-level ozone | -0.35 (-4 to 3.43) | -1.88 (-4.72 to 1.04) |
| Artificial surface | -0.35 (-3.79 to 3.22) | 5.72 (2.54 to 9) |
| Population density | 0.02 (-2.41 to 2.52) | 2 (-0.08 to 4.13) |
| Rural population | 0.58 (-3.16 to 4.46) | -5.64 (-8.57 to -2.62) |
| Houses built before 1971 | -0.14 (-3.9 to 3.76) | 2.6 (-0.66 to 5.97) |
| Houses with central heating | -0.4 (-4.09 to 3.42) | 5.72 (2.49 to 9.05) |
| Home ownership | 3.1 (-0.5 to 6.83) | -4.94 (-7.76 to -2.02) |
| People over 64 years | -0.38 (-4.17 to 3.56) | -4.46 (-7.56 to -1.25) |
| People over 64 years living alone | -2.86 (-6.3 to 0.71) | 4.17 (1.1 to 7.33) |
| People over 24 years without primary education | 0.43 (-3.24 to 4.24) | 1.14 (-2.12 to 4.51) |
| Median income by consumption unit | 0.21 (-3 to 3.51) | 2.05 (-0.83 to 5.01) |
| Households with 2 or more cars | 1.92 (-1.68 to 5.66) | -5.18 (-8.03 to -2.25) |
| Unemployment rate | 0.16 (-3.25 to 3.69) | 1.74 (-1.17 to 4.74) |
| Votes for the right | -0.45 (-4.16 to 3.39) | 0.35 (-2.8 to 3.59) |

**Table S3. Effect modifiers of heat-related mortality risk after adjustment for nitrogen dioxide**

The table reports the %CR of mortality at the 99th percentile of daily temperature between the 10th and 90th percentiles of each contextual indicator after adjustment for nitrogen dioxide.

| | Variable | Nitrogen dioxide |
|---|---|---|
| Temperature | 0.28 (-2.52 to 3.17) | 8.33 (4.83 to 11.95) |
| Relative humidity | 2.78 (-0.5 to 6.17) | 9.76 (5.99 to 13.67) |
| Particulate matter ≤ 10 μm | 0.84 (-2.65 to 4.47) | 7.71 (3.46 to 12.13) |
| Particulate matter ≤ 2.5 μm | -0.76 (-3.44 to 2) | 9.15 (5.29 to 13.16) |
| Ground-level ozone | -3.06 (-5.86 to -0.16) | 8.96 (5.47 to 12.57) |
| Artificial surface | 0.79 (-3.02 to 4.74) | 7.8 (3.54 to 12.23) |
| Population density | 0.88 (-1.29 to 3.1) | 6.39 (2.47 to 10.46) |
| Houses built before 1971 | 1.33 (-1.92 to 4.68) | 8.2 (4.8 to 11.72) |
| Houses with central heating | 2.94 (-0.37 to 6.36) | 7.19 (3.65 to 10.85) |
| Home ownership | -1.6 (-4.82 to 1.73) | 7.49 (3.69 to 11.42) |
| People over 64 years | -2 (-5.35 to 1.47) | 6.57 (2.89 to 10.37) |
| People over 64 years living alone | 1.78 (-1.34 to 4.99) | 7.75 (4.2 to 11.42) |
| People over 24 years without primary education | 0.65 (-2.59 to 4) | 7.38 (3.86 to 11.01) |
| Median income by consumption unit | -0.44 (-3.37 to 2.57) | 8.68 (5.15 to 12.33) |
| Households with 2 or more cars | -1.6 (-4.89 to 1.82) | 7.45 (3.6 to 11.43) |
| Unemployment rate | 1.14 (-1.76 to 4.12) | 8.31 (4.89 to 11.84) |
| Votes for the right | -1.21 (-4.33 to 2.01) | 8.62 (5.18 to 12.17) |
| Rural population / Nitrogen dioxide | -1.59 (-5.09 to 2.05) | 7.4 (3.43 to 11.52) |

**Figure S5. Relationship between temperature and minimum mortality temperature**

The figure represents the relationship between average temperature and minimum mortality temperature (MMT). Bullet points represent spatial units.

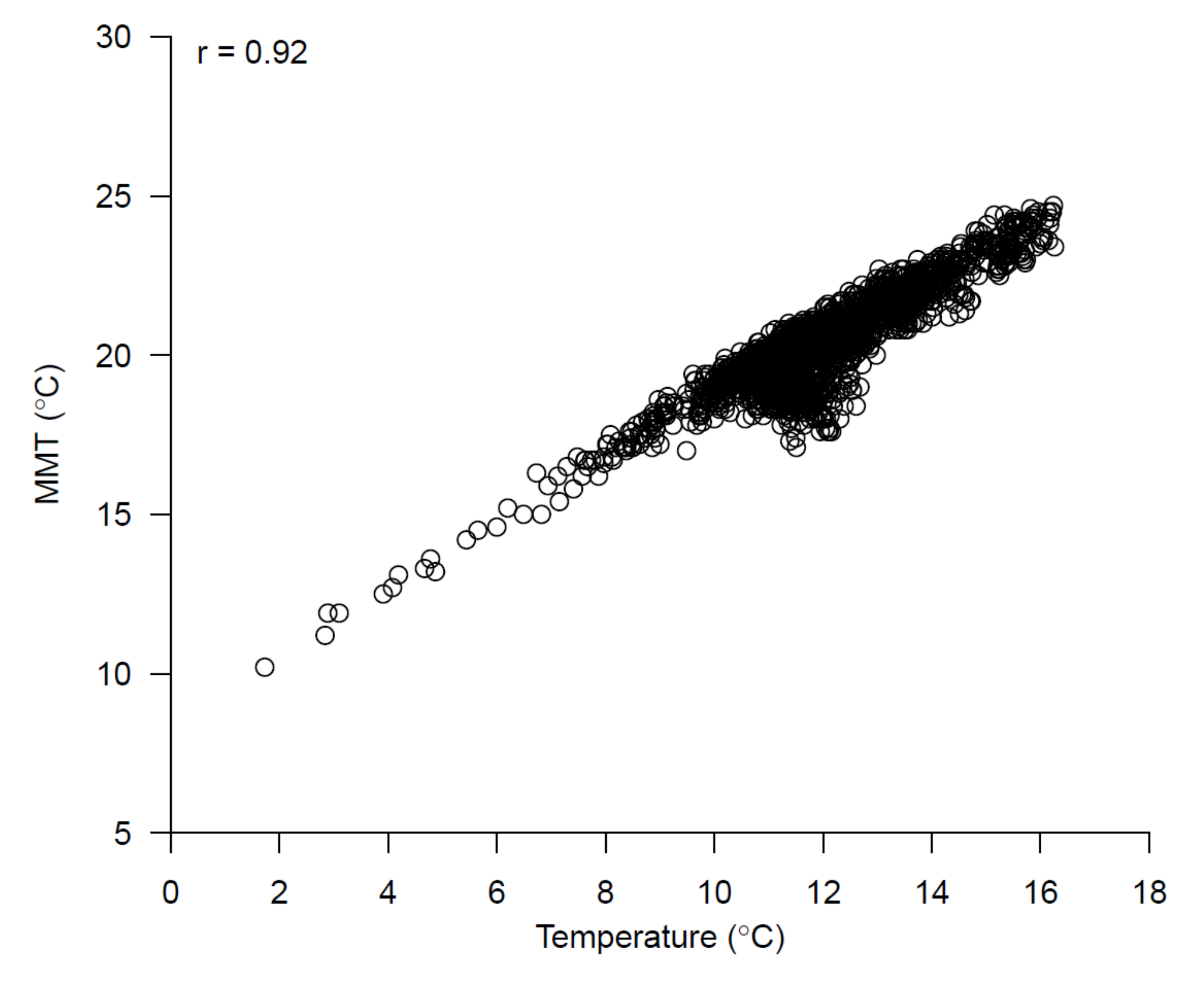

**Figure S6. Ratio of temperature-related mortality risk between high and low air pollution days**

Ratio of relative risk (RRR) of temperature-related mortality with regard to the minimum mortality temperature (MMT) between high (above canton-specific median) and low (below province-specific percentile) air pollution days. Shaded areas represent the 95% CI.

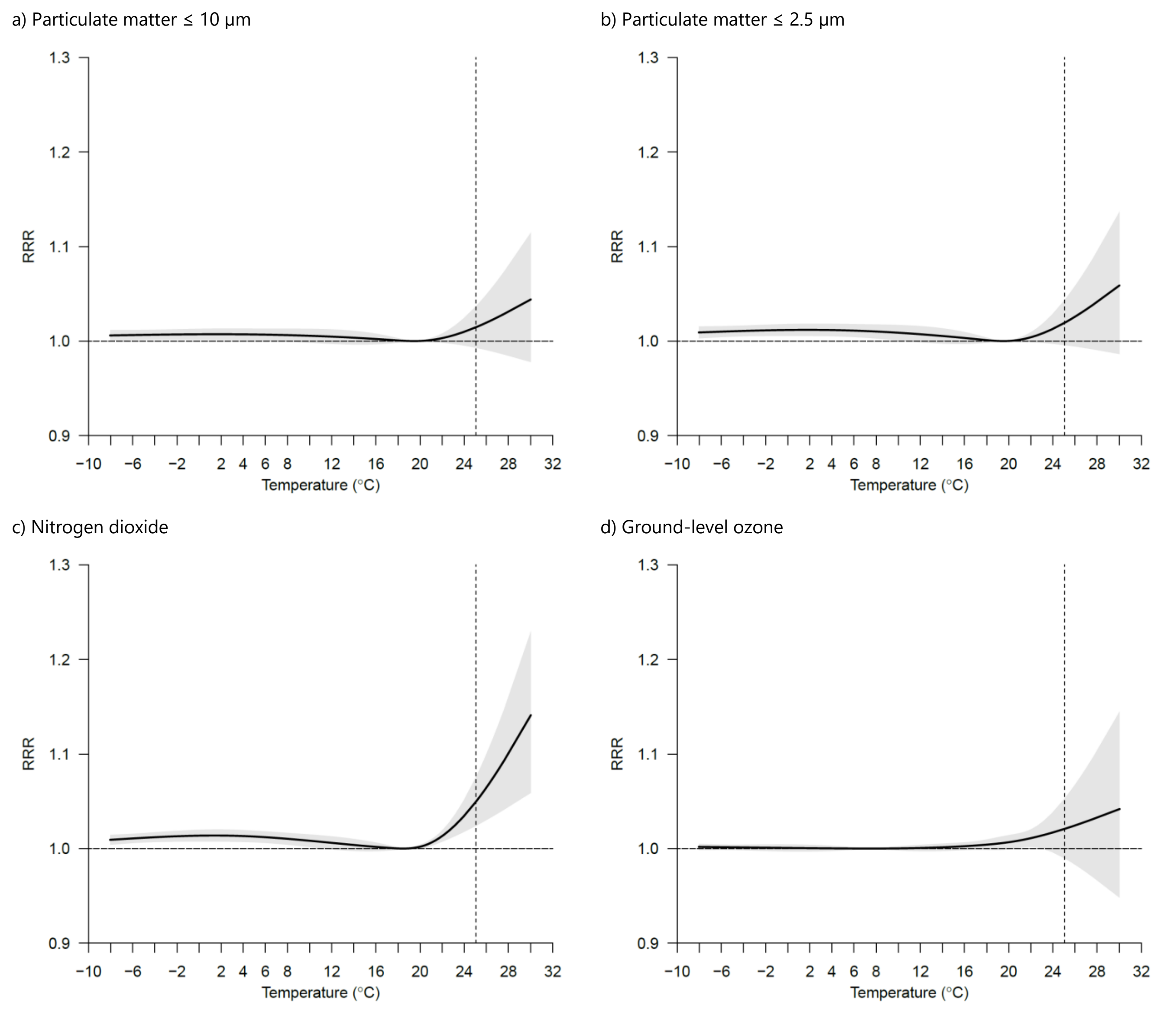


**Description of the statistical analysis:**
To assess the effect modification of heat by air pollutants, we included separately in the time-series quasi-Poisson regression model an interaction between the cross-basis function of temperature and dummy variables representing air pollution categories [low (below the pseudo-canton-specific median) and high (above the pseudo-canton-specific median)], and a linear term for the air pollutants in order to account for potential residual confounding.
